# Reorganization increases long-term sickness absence at all levels of hospital staff: panel data analysis of employees of Norwegian public hospitals

**DOI:** 10.1186/1472-6963-14-411

**Published:** 2014-09-19

**Authors:** Mari H Ingelsrud

**Affiliations:** Department of Social Work, Child Welfare and Social Policy, Oslo and Akershus University College of Applied Sciences, Post box 4, St. Olavs plass, N-0130 Oslo, Norway

**Keywords:** Sickness absence days, Sick leave, Organizational change, Register data, Fixed effects Poisson regression, Hospital, Norway

## Abstract

**Background:**

The Norwegian specialist health service has undergone many processes of reorganization during the last three decades. Changes are mainly initiated to increase the efficiency and quality of health care serving an ageing population under the condition of a diminishing labour supply. The aim of this study is to investigate the effects of reorganization on long-term sickness absence among different levels of hospital staff.

**Methods:**

The study draws on panel data on employees of Norwegian public hospitals in 2005 and 2007 (N = 106,715). National register data on individual employees’ days of medically certified long-term (>16 days) sickness absence were linked with survey measures of actual reorganization executed at each hospital in each year. The surveys, answered by hospital administration staff, measured five types of reorganization: merging units, splitting up units, creating new units, shutting down units and reallocation of employees. The variation in sickness absence days was analysed using random and fixed effects Poisson regression with level of reorganization as the main explanatory variable.

**Results:**

The fixed effects analysis shows that increasing the degree of organizational change at a hospital from a low to a moderate or high degree leads to an increase in the number of days of long-term sickness absence of respectively 9% (95% CI: 1.03-1.15) and 8% (95% CI: 1.02-1.15). There are few significant differences between employees in different education categories. Only physicians have a significantly higher relative increase in days of long-term sickness absence than the control group with lower tertiary education.

**Conclusions:**

Increased long-term sickness absence is a risk following reorganization. This risk affects all levels of hospital staff.

## Background

Restructuring is increasingly being employed as a management strategy in the public sector, and consequently, in the health sector all over the world. Additionally, in Norway the specialist health service has undergone major changes since the mid-1990s. These have mainly been initiated to increase efficiency and quality in the health services to meet the challenges of an ageing population and diminishing labour supply [1]. The impact of restructuring on the health of employees is not fully understood and more research is needed [2]. This study contributes to the field by investigating the effects of reorganization on the number of days of long-term sickness absence taken by employees at all levels of the Norwegian public hospitals.

Earlier studies of the effects of restructuring on health, sickness absence and morbidity have mostly focused on downsizing and downsizing survivors, with inconclusive results. Downsizing has been shown to increase sickness absence is some studies [3, 4], but others find no such effect [5], or even find sickness absence to decrease [6]. Several studies have revealed that restructuring not involving lay-offs can have a detrimental effect on health [4, 7], and in one study reorganization was more associated with health problems than downsizing [8]. Few studies have examined the effects of reorganization on sickness absence for different sectors and different groups of employees, leaving a need for more research into whether the effects of reorganization on employee health are unequally distributed [9].

There are a number of studies of reorganization and sickness absence in the health services: Røed and Fevang [7] studied nurses in Norway. As layoffs are rare in the Norwegian public sector, downsizing was used as a proxy for organizational change. They found that a 20% or higher reduction in hours of work at a workplace led to increased sickness absence rates among the remaining employees. A study of health professionals employed in Norwegian hospitals found that the risk of long-term sickness absence was related to the frequency of structural changes at the hospital, but not to patient-related changes [10]. In the Finnish hospital sector, the privatization of laboratory and radiology units was not associated with increased long-term sickness absence [11].

A systematic review of the health effects of task reorganization with reference to the demand-control model [12] sheds light on two possible mechanisms; the review concluded that changes that decreased control and increased demand had adverse health effects [9]. Studies of restructuring in the Canadian health sector found that reorganization and downsizing were associated with higher work demands [13]. Lower decision latitude following reorganization and downsizing was associated with higher sickness absence among nurses [14]. Less time to plan work following a reorganization process at a large teaching hospital in Sweden was associated with increased long-term sickness absence across all employees [15]. In light of the existing research, the main hypothesis of this paper is that the net effect of reorganization is increased long-term sickness absence among employees.

The study combines individual level register data on sickness absence spells longer than 16 days with hospital level survey data on organizational change. Due to the limitations of the data, it is not possible to disentangle the mechanisms at work in this study. It is however possible to investigate if different occupational categories of employees are affected differently. The consequences of reorganization at a hospital may vary greatly between occupations. Earlier research has shown that there is a social gradient in working conditions and health where the lowest occupational category has both the lowest degree of control at work, the worst health and the highest level of sickness absence [16]. The combination of low control and high demands is thought to be the most detrimental to health [12]. The effect of reorganization is thus likely to be more severe for those with the lowest education. However, the lower level staff may not be very involved in the hospital’s reorganization processes [17] and may not therefore be affected by the changes to the same degree as the higher level staff. There are conflicting theories with respect to which employee categories are likely to be most affected by reorganization. The second aim of this article is to investigate how long-term sickness absence among different levels of hospital staff is affected by the reorganization.

## Methods

### Data

The register based employment statistics of Statistics Norway was used to link individual information on the employees to organizational characteristics of the hospital where they work. The target population was all employees of the Norwegian public hospitals in the third week of November in 2005 and 2007. Individual data was collected from the national register data containing demographic information, information about work, education and welfare receipt, including sickness absence spells of more than 16 days, from 1992 to 2008.

The organization of the Norwegian specialist health sector has been tracked by biannual surveys sent to all specialist hospitals in Norway since 1999. Questions about organizational changes made during the last 12 months were added to the questionnaire in 2005. This paper analyses data from 2005 and 2007. In 2005, 52 out of 63 (83%) public hospitals answered the questions about reorganization. Between 2005 and 2007 several hospitals were merged, lowering the total number of hospitals surveyed. In 2007, 52 out of 57 (91%) public hospitals answered the same questions. 48 hospitals answered the questions in both years. Only employees who were employed in the same job for the whole year in 2005 or 2007 were included, leaving a sample of 106,715 observations (person-years).

All hospitals in Norway have a surgical and medical field of operation (divisions), either localized in distinct units or sharing some units, for example hospital beds. In cases where there was more than one medical or surgical division, or where the divisions were in separate locations, the hospitals were asked to answer one questionnaire per division. A department manager usually answered the survey. Most of the hospitals (60-80%) returned one questionnaire each for the medical and surgical operational divisions. Some only returned questionnaires for one of the operational divisions (15-30%), and even fewer (3-12%) returned questionnaires from between 3 and 6 different operational divisions. As there is no record of where in the hospital each employee works, information from the surgical and medical divisions was aggregated to the hospital level before merging with individual records. This study can be understood as investigating the net effects of concrete organizational changes at a hospital.

The study was approved by the Regional Committees for Medical and Health Research Ethics (REK) and the Norwegian Social Science Data Services (NSD).

### Reorganization

The types of reorganization measured in this survey were 1) merging units, 2) splitting up units, 3) creating new units, 4) shutting down units and 5) reallocation of employees. The questionnaire asked for the number of changes in the last twelve months prior to the questionnaire being answered. For 2005 this therefore covers changes in the hospitals from autumn 2004 to autumn 2005. The 2007 data covers changes in the hospital from autumn 2006 to autumn 2007. Response options were "no", "yes, once" and "yes, more than once". These responses were recoded into values 0, 1 and 2. The questions were recorded for the medical and surgical divisions, and aggregated to the hospital level as average times the change occurred per division. It is likely that many changes are more stressful than one change. As a proxy for degree of reorganization, the total number of the organizational changes is used. The variable has a theoretical span from 0: "no changes in any of the hospital’s divisions" to 10: "more than one change of each type in all of the hospital’s divisions", but the average number of changes reported in 2005 and 2007 were 2.2 and 2.8 reorganizations per division. The variable is divided into a dummy set of 3 variables: low degree (on average 1.5 or less), moderate degree (on average 1.5 to 3) and high degree of reorganization (on average more than 3).

### Sickness absence

The outcome variable is the yearly aggregated number of days of sickness absence spells lasting longer than 16 days. In the Norwegian public sector sickness absence is fully reimbursed from the first day of absence, with a maximum duration of one year. The first 16 calendar days of absence are paid by the employer. Benefits from the 17th day are paid by the national health insurance and only sickness absence spells of this length and above are recorded in the register data. Sickness absence is recorded for the calendar years 2005 and 2007 as number of days per year, including the 16 days that are covered by the employer.

### Education

Education is often used as a measure of socioeconomic status, along with income and occupation, and has a strong correlation with disease [18]. Every citizen’s education is registered using the Norwegian Standard Classification of Education. The register has information on the highest completed level of education, including the specific field. Two major professions in the specialist health sector, physicians and nurses, are possible to identify from the data because they require specific vocational training. The specific occupation of other health staff can also identified based on field of training. However, for many of the employees in the hospitals, for example in the administration, it is not possible to identify specific occupations based on field of education. Education level is coded into seven dummy variables based on both level and field of education: Primary education (up to 10 years of school), secondary health training (up to 14 years), other secondary education (11 to 14 years), trained nurses (lower tertiary college degree), other lower degree tertiary education (up to four years of university or college education), physicians (higher tertiary university degree) and other higher degree tertiary education (five years or more in university or college).

### Control variables

Gender is coded as male (1) and female (0). Age is coded in years. In the fixed effects analyses, ageing and the passing of time are perfectly collinear. Thus, it is not possible to distinguish between ageing and time effects, and they are in effect included in the same variable.

### Statistical methods

In order to investigate the effects of reorganization on sickness absence, the variation in sickness absence and level of reorganization in the employee’s workplace was analysed with panel data models. Panel data models are more efficient than OLS, because they exploit the longitudinal structure of the data while controlling for the serial correlation in errors that is a consequence of the same individual occurring twice in the data. The dependent variable, number of days of long-term sickness absence, is count data. These types of data are best modelled with Poisson regression. Random effects Poisson regression provides the most efficient estimator of the effect of reorganization on long-term sickness absence, utilizing both the variation between employees and within employees over time [19].

However, selection is an issue when studying the effects of reorganization on health, as the workplaces or employees susceptible to reorganization might be different on unobserved characteristics like general health. Random effects models, like cross-sectional studies, do not control for this type of omitted variables bias.

The panel structure of the data allows for consistent estimation of the fixed effects model. Fixed effects Poisson models are used to analyse how changes in the amount of reorganization and changes in sickness absence vary within the same person from 2005 to 2007. This eliminates the omitted variables bias caused by the time-constant unmeasured characteristics that differ between hospitals and employees [19]. In the case where there is no selection, the fixed and random estimators are the same. This is tested with a Hausman test [19].

One disadvantage of the fixed effects model is that only coefficients for variables that vary over time can be estimated. Another disadvantage of the fixed effects Poisson model is that respondents without a change in the number of days of long-term sickness absence from 2005 to 2007 are dropped from the analysis [19]. The practical consequence is that the fixed effects results are based on the employees that have some sickness absence in at least one of the years, leaving out those that are the most healthy. If the model is correctly specified and the effects are constant throughout the population, this does not present a problem, other than the lowered sample size widening the confidence intervals. However, it is not given that the effect of restructuring is constant across the population. This assumption is to some degree tested by running random effect regressions on both the total sample and the fixed effects sample, checking for differences in average effect sizes between the two samples. As another sensitivity test, I have run the models using linear fixed effects regression (not shown), which includes all respondents with data for both years. The linear fixed effects models display similar results to the Poisson regressions, and are not commented upon further.

The interpretations of the two models are different: the fixed effects estimators show how much the number of days of long-term sickness absence increases from one year to the next if the level of reorganization in an employee’s workplace changes from low to moderate, or low to high. The random effects model uses the optimal combination of within and between variation, so the estimators also include information on how much higher the number of days of long-term sickness absence are for employees at hospitals with a moderate or high level of reorganization compared to employees at hospitals with a low level of reorganization. Any selection of workers into hospitals that are more prone to reorganize is not controlled for in random effects. By running both the random and fixed effects models, the study takes advantage of each model’s merits.

Fixed and random effects Poisson regression models are first run on a base model to investigate the effect of reorganization across all employees. Interaction effects between reorganization and the dummy variables for education are then included to test whether the effects of reorganization are different for different education categories. The regression analyses are conducted using the xtpoisson command with FE and RE options in Stata 12. All models are run with the vce(bootstrap) option with 400 repetitions to obtain cluster robust standard errors [20].

## Results

### Descriptive statistics

Descriptive statistics for the sample are presented in Table 1. There is a strong social gradient in the distribution of long-term sickness absence days. The lowest educated group has on average 39 days of long-term sickness absence per year, while physicians have an average of 9.4 days. The differences reflect the social gradient in health [16]. The average number of days of long-term sickness absence per year rose from 23.3 in 2005 to 24.8 in 2007. This is in accordance with other statistics on sickness absence in the Norwegian health sector, showing a rise from 2005 to 2007 [21]. The quite large standard deviations for the sickness absence statistics show that the average number of days of long-term sickness absence is elevated by the few employees with very long (limited to one year) sickness absence spells.

**Table 1:** **Descriptive statistics by education and year for the sample used in the random effects analyses**

	Sample			Sickness absence, days (spells of 16 days or longer)		Age			Reorganization		
	N	%	% included in FE analysis	Mean	SD	Mean	SD	Male	Low (<=1.5)	Moderate (1.5-3)	High (>3)
Total											
2005	55,933		28%	23.3	57.3	43.9	11.0	19%	39%	35%	26%
2007	50,782		31%	24.8	59.9	44.9	11.0	19%	16%	52%	31%
Baseline characteristics in 2005 by education											
Primary	4,048	7%	35%	39.0	75.3	47.7	10.1	18%	·	·	·
Secondary	7,463	13%	30%	26.5	61.6	47.5	10.9	24%	·	·	·
Secondary health training	6,830	12%	38%	32.0	66.7	47.1	11.1	7%	·	·	·
Tertiary, lower	9,376	17%	25%	19.3	52.0	42.5	10.6	20%	·	·	·
Trained nurses	21,608	39%	28%	22.1	54.5	41.4	10.5	8%	·	·	·
Tertiary, higher	1,702	3%	15%	14.1	42.7	41.5	10.3	39%	·	·	·
Physicians	4,906	9%	14%	9.4	35.6	45.3	10.7	66%	·	·	·

### Multivariate analysis

Results from the base models are shown in Table 2. Both random and fixed effects models were run. The results of a Hausman test showed that the estimators in the two models are significantly different (chi-square: 7915, p<0.001), and that the random effects estimators are biased [19]. To test if there is any selected sample bias due to the fact that the fixed effects model is run on only the most absent part of the sample, random effects Poisson regressions were done for both the full sample and the fixed effects sample (not shown). The coefficients for reorganization are somewhat higher when random effects is run on the fixed effects sample than on the full sample (in the analysis presented in Table 2 the coefficient for a moderate degree of reorganization increases from 0.13 to 0.17. The coefficient for a high degree increases from 0.11 to 0.14). This may be understood as there being some sample bias, where the effects of restructuring on sickness absence may be somewhat higher for the included sample than for the rest of the population. Nonetheless, the fixed effects coefficients of reorganization are smaller than the random effects, so the most sober estimates are commented upon in this article. Looking at the fixed effects estimates, employees at hospitals that increased the degree of reorganization from low to moderate from 2005 to 2007 have had a 9% increase in number of sickness absence days in the same period. Increasing the degree of reorganization from low to high from 2005 to 2007 led to an 8% increase in sickness absence spells. The base models in Table 2 have been run for both employees over the age of 54 and for those that are 54 years or younger (not shown). Although the coefficients for reorganization for those over 54 years are higher than for those that are 54 years or younger, the confidence intervals are overlapping, and the effects of reorganization are still significant for the age group under 54 years.

**Table 2:** **The effect of reorganization on number of days of long-term sickness absence**

	Change in number of days		Change in number of days	
	Random effects		Fixed effects	
	Coeff. IRR	95% CI	Coeff. IRR	95% CI
Education: Reference cat. Tertiary, lower degree				
Primary	1.50***	1.35-1.67	·	·
Secondary	1.16*	1.04-1.29	·	·
Secondary health training	1.38***	1.26-1.52	·	·
Trained nurses	1.38***	1.28-1.49	·	·
Tertiary, higher degree	0.76**	0.63-0.91	·	·
Physicians	0.51***	0.45-0.58	·	·
Age	1.11***	1.09-1.12	1.14***	1.12-1.16
Male	0.42***	0.39-0.46	·	·
Reorganization (Low as reference)				
Moderate	1.13***	1.06-1.20	1.09**	1.03-1.15
High	1.11**	1.05-1.18	1.08**	1.02-1.15
Constant	0.41**	0.25-0.68	·	·
N (Person-years)	106,715		31,324	
N (persons)	68,630		15,662	

Note: Poisson regression. Incidence Rate Ratio shown with 95% confidence intervals using robust standard errors computed with vce(bootstrap). Significance probabilities (***p < .001, **p < .01, *p < .05).

The results of the random and fixed effects Poisson regression models including the interaction effects for education are shown in Table 3. The coefficients in the random effects model are slightly higher than in the fixed effects model, but the main findings are the same. The F-test of the interaction terms between education category and the reorganization dummy set in the fixed effects model shows that the differences in effect size between the categories are just above the level of statistical significance (p = .058). The interaction terms are barely significant in the random effects model (p = .032). Only physicians have a significantly higher relative effect of a high degree of reorganization on sickness absence than the reference group with lower tertiary education. This indicates that employees at all levels of the hospitals are affected by the organizational changes.

**Table 3:** **The effect of reorganization on sickness absence - education interaction effects**

	Change in number of days		Change in number of days	
	Random effects ^a^		Fixed effects	
	Coeff. IRR	CI	Coeff. IRR	CI
Age	1.10***	1.09-1.12	1.14	1.12-1.16
Reorganization (low degree as reference)				
Moderate (Tertiary, lower)	1.15	0.98-1.34	1.11	0.95-1.29
High (Tertiary, lower)	1.01	0.86-1.19	0.99	0.85-1.16
Primary × moderate	0.85	0.68-1.07	0.85	0.67-1.08
Primary × high	1.13	0.88-1.45	1.12	0.87-1.43
Secondary × moderate	1.01	0.83-1.23	1.01	0.82-1.25
Secondary × high	1.13	0.92-1.39	1.13	0.91-1.39
Secondary health training × moderate	0.99	0.80-1.22	0.99	0.82-1.20
Secondary health training × high	0.99	0.80-1.22	0.99	0.80-1.21
Trained nurses × moderate	1.01	0.85-1.19	1.01	0.85-1.20
Trained nurses × high	1.12	0.93-1.35	1.12	0.94-1.34
Tertiary, higher degree × moderate	0.96	0.57-1.63	0.96	0.59-1.56
Tertiary, higher degree × high	1.41	0.78-2.56	1.40	0.81-2.45
Physicians × moderate	1.08	0.78-1.50	1.07	0.78-1.46
Physicians × high	1.51*	1.05-2.19	1.50*	1.05-2.14
N (Person-years)	106,715		31,324	
N (Persons)	68,630		15,662	

Note: Poisson regression. Incidence Rate Ratio shown with 95% confidence intervals using robust standard errors computed with vce(bootstrap). Significance probabilities (***p < .001, *p < .05).

^a^Gender and main effects for education were included in the random effects model.

## Discussion

The analyses show that increasing the degree of reorganization (merging units, splitting up units, creating new units, shutting down units and reallocation of employees) at a hospital leads to an increase in sickness absence. This is in line with previous research showing that reorganization leads to work-related health problems [8] and sickness absence [4, 7]. Theoretically relevant mechanisms by which reorganization leads to higher sickness absence are increased demands on employees, reduced sense of control, increased job insecurity, or a combination of these. How each of these mechanisms contributes to the effect of reorganization is outside the scope of this article, but it has been shown here that the net effect of increased reorganization is higher sickness absence.

Due to the fact that organizational change is a hospital level measure, the variation between employees is limited. This leads to large standard errors and low explained variance. The results show that employees at all levels of the hospitals are affected by reorganization. Only physicians have a significantly higher effect of a high degree of reorganization, compared to the reference category. The analysis does not support the claim that reorganization leads to relatively more sickness absence for the lowest educated employees. However, it is important to point out that even though the relative effect of reorganization on sickness absence is not significantly higher for those with primary education, the confidence intervals are wide. This is also the group with the most sickness absence days, and the social gradient in sickness absence [16] is still striking after reorganization is taken into account.

There are several possible explanations for these results; one explanation is that employees in different education categories might be affected by organization changes to different degrees. The results might reflect that the lowest educated employees are less involved in the reorganizations that take place at a hospital than the higher educated employees. Maybe they are only aware of the changes that affect their specific work unit, while the higher educated employees are more involved in the whole reorganization process right from the initial planning. Responsibilities concerning the effects of the organizational changes are also less likely to lie with the lowest educated. The increase in demands and/or loss of control during a restructuring process might therefore be greatest for the highest educated. A qualitative study of the reorganization processes at one Norwegian teaching hospital found that physicians in particular, but also nurses were stretched between the demands of giving good care and administrative involvement in the planning, building of and moving to a new hospital [22]. Another study of the same hospital reorganization reported that the lower level staff were less involved in the reorganization processes [17]. The perceived loss of control during a reorganization process might also be greater for the higher educated, as they are used to having a relatively high degree of control compared with lower level employees. The highest educated may experience some sort of relative deprivation [23] when they compare the amount of control and demands that they encounter at work to what they were used to before the reorganization process started, or to the level encountered by colleagues at hospitals that are not reorganizing.

Employee attendance, or conversely, absenteeism, is likely to be influenced by both motivation and ability to attend work [24]. Even though the long-term sickness absence measured in this article is physician certified, it is important to draw a distinction between disease and sickness absence. In addition to disease, sickness absence is dependent on personal attitudes, social attitudes and the physician’s comprehension of the situation [25]. Analyses of data from the UK Whitehall II study of civil servants showed that absence spells longer than seven days were more strongly correlated with self-reported health, long standing illness and other measures of health than shorter sickness absence spells [26]. As only longer spells of absence are registered in this study’s data, it is likely that absence spells that are largely motivation-related, are at a minimum in these data. However, the length of an absence spell may be prolonged by motivation factors, as well as an impaired state of health. The social and motivation-related aspects of sickness absence must be kept in mind, and the results of these analyses cannot solely be attributed to the individual’s impaired state of health.

### Strengths and limitations

The register data used in these analyses are a detailed, unbiased longitudinal source of information on all employees of Norwegian public hospitals. However, many variables are not included. A limitation of the study is the lack of information concerning the mechanisms by which reorganization leads to sickness absence: job insecurity, rewards, demands, control and other measures of psycho-social work characteristics are not recorded in the data. Neither is health behaviour or other measures of health.

The administrative data source of reorganization ensures that there is no personal bias in the reports. It is unfortunate that reorganization is not recorded per employee. This is likely to give underestimated measures of the effect of reorganization on sickness absence, since it is not possible to differentiate between employees at the same hospital who are personally affected by different amounts of reorganization.

The longitudinal nature of the data allows for the use of fixed effects analysis to control for unobserved time-constant characteristics among the employees. It does not, however, control for unobservable characteristics that vary over time. This can be changes in HRM policies or management at the hospitals. To the extent that these covary with both sickness absence and reorganization, the estimates from the fixed effects regression can be biased upward. The estimates must not be interpreted as pure causal effects of reorganization, as all unobserved heterogeneity might not be factored out.

A characteristic of the fixed effects Poisson model is that only employees with a change in the dependent variable from 2005 to 2007 are included in the analyses, excluding those with no long-term sickness absence in any of the years. This increases the standard errors, but might also limit the generalizability of the estimator if the effects are not constant throughout the population. The analyses suggest that there is some sample bias, where the effects of reorganization on sickness absence might be somewhat higher for the included sample than the rest of the population.

The aim of this study was to analyse the effect of reorganization on sickness absence in Norwegian public hospitals. In this part of the public sector reorganization does not usually include lay-offs [27]. The Norwegian health sector is characterized by a high proportion of female employees, shift work and a high incidence of part-time work [21]. Furthermore, unemployment is very low in Norway and social security benefits are quite generous. These characteristics let us study the effects of reorganization on sickness absence separate from effects of unemployment and fear of being laid off. The effects of reorganization in the Norwegian labour market are likely to be different than in other labour market contexts. These findings may therefore not be directly generalized to other countries and markets. Still, research on the effect of reorganization on sickness absence is needed in various labour market contexts, and in both the private and public sector. A comparative analysis of the effects of reorganization in various labour market contexts may be informative as to which types of mechanisms are in play.

## Conclusion

This study suggests that reorganization has a detrimental effect on long-term sickness absence among employees at all levels of the hospital. Consequences of reorganization for the health of employees should be considered before changes are launched, as increased sickness absence is likely to be one of the costs.
